# Red cell distribution width and erythrocyte osmotic stability in type 2 diabetes mellitus

**DOI:** 10.1111/jcmm.16184

**Published:** 2021-02-16

**Authors:** Maria Aparecida Knychala, Mario da Silva Garrote‐Filho, Breno Batista da Silva, Samantha Neves de Oliveira, Sarah Yasminy Luz, Manuela Ortega Marques Rodrigues, Nilson Penha‐Silva

**Affiliations:** ^1^ Institute of Biotechnology Federal University of Uberlândia Uberlândia Brazil

**Keywords:** erythrocyte osmotic resistance, haemoglobin, iron, osmotic fragility test, red cell distribution width, red blood cells, T2D

## Abstract

This study aimed to investigate the relationship between red cell distribution width (RDW) and erythrocyte osmotic stability in non‐diabetic and diabetic individuals in both sexes. The study sample (N = 122) was constituted by 53 type 2 diabetics (DM) and 69 non‐diabetics (ND), being 21 and 22 men in each group, respectively. The osmotic stability of erythrocytes was obtained by the variation in saline concentration (dX) capable of determining hypoosmotic lysis. Higher RDW values and lower serum iron concentrations were found in the diabetic group when compared to the non‐diabetic volunteers. In the group of diabetic women, RDW was positively correlated with the reticulocyte index, and both RDW and dX were negatively correlated with iron, haemoglobin, transferrin saturation index, mean corpuscular haemoglobin and mean corpuscular haemoglobin concentration. In all the groups studied, RDW was positively correlated with dX, especially in the diabetic group, where the correlation was the strongest. RDW elevation in both women and men with type 2 diabetes mellitus was associated with decreased serum iron indicators. Furthermore, RDW has a similar meaning to dX, as small erythrocytes have less haemoglobin, resulting in both an increase of RDW and dX.

## 1 INTRODUCTION

Hyperglycaemia, present in type 2 diabetes mellitus (T2D), has diverse effects on erythrocytes, far beyond the known process of haemoglobin glycation. These effects include a decrease of both deformability and fluidity^1^, ^2^, ^3^ and lifespan^4^, ^5^ of these cells, in addition to increased adhesion.^6^

The decreased erythrocyte deformability in T2D has been attributed to increased blood glucose, glycations, oxidative stress and increased content of saturated fatty acids in its membrane.^7^, ^8^ Less deformable erythrocytes would contribute to the development of T2D‐associated vascular complications.^9^, ^10^

One of the manifestations of the lower erythrocyte deformability would be increased volume variability,^11^ which is given by the red cell distribution width (RDW), a haematological index determined in a complete blood count. Indeed, the retrospective study of Nada and colleagues found that RDW is higher in diabetic than in non‐diabetic individuals.^12^ This makes sense, as elevated RDW values have been associated with diseases in which inflammatory status is elevated.^13^, ^14^, ^15^, ^16^, ^17^, ^18^

The present study further investigates the relationship between diabetes and RDW, using a transversal approach in the evaluation of blood samples of female subjects without diabetes, or T2D. Important biochemical and haematimetric variables and erythrocyte osmotic stability parameters were analysed.

The evaluation of erythrocyte osmotic stability is very important for the study of these cells^19^, ^20^, ^21^, ^22^, ^23^, ^24^, ^25^ and constitutes a relevant clinical tool, not only for the characterization of erythrocytopathies, but also for the study of several diseases, such as type 1 diabetes,^26^ malaria and pre‐eclampsia^27^, ^28^ and clinical interventions such as bariatric surgery^29^ and physical exercise^30^ among other situations that may affect red blood cells.

As far as we know, the present study is the first to investigate the causes of higher RDW value in type 2 diabetes, with important contributions about this issue.

## 2 MATERIALS AND METHODS

### 2.1 Population

**Figure 1:**
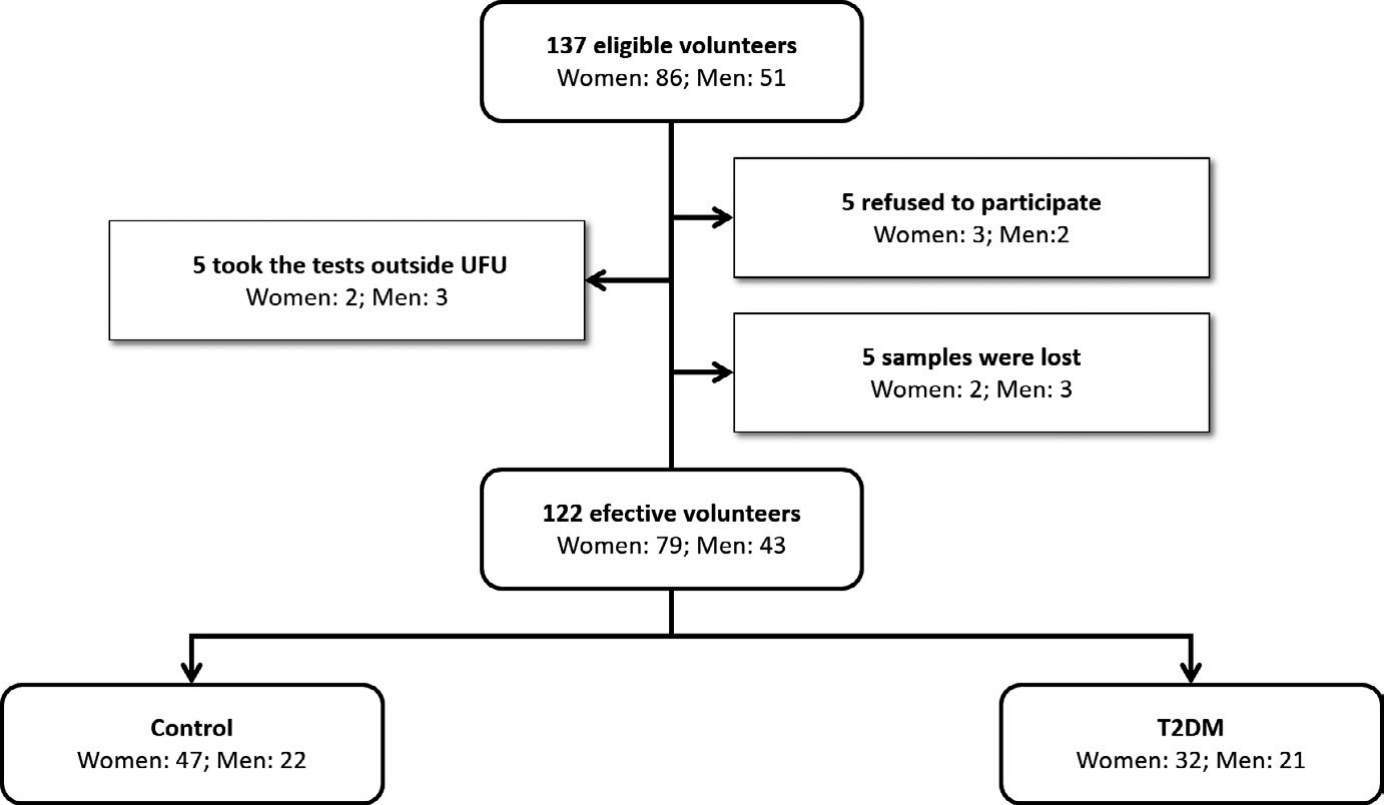
Flow chart of volunteers of the study

This study was previously authorized by the Research Ethics Committee of the Federal University of Uberlandia (UFU) under registry # 56557516.0.0000.5152. The study sample had 122 individuals in all, 53 being type 2 diabetics (T2D) and 69 non‐diabetics (ND). The male amount in each group was 21 and 22, respectively (Figure 1). All volunteers were treated at the outpatient clinic of the UFU Clinical Hospital, dully classified according to the diagnostic criteria. The following criteria were used for diabetes diagnosis: fasting plasma glucose ≥ 126 mg/dL (7.0 mmol/L) or HbA1c ≥ 6.5% (48 mmol/mol). The criteria used for non‐diabetics were fasting plasma glucose ≤ 99 mg/dL (5.5 mmol/L) or HbA1c ≤ 5.6% (38 mmol/mol). These criteria were established by the American Diabetes Association.^31^ At first, the fasting was 8 hours, but it was considered a 12 hours fasting for cholesterol measurement.

Among the patients eligible to participate in the study, those with cognitive impairment and hereditary erythrocytopathies, as well as a history of abuse of alcohol and other drugs, were not included. Patients who had signed the informed consent form and decided to abandon the study, regardless of the reason, were excluded.

The diabetic patients took drugs commonly used by those affected by this disease. Such medications include: metformin from 500 to 2000 mg; statins such as simvastatin (20 to 40 mg) and rosuvastatin (10 to 20 mg); antihypertensives such as losartan or atenolol (25 to 100 mg) or enalapril (20 to 40 mg); aspirin at 100 mg; and insulin from 20 to 40 UI. Besides that, all participants with a proven vitamin B12 deficiency, especially those using metformin, received vitamin B12 supplementation with pills of 3000 or 5000 mcg.

We considered the value of HbA1c of 7% as an indicator of good glycaemic control and a value of 6.5% in the diagnosis of T2D. We also established a cut‐off of 7% at HbA1c to compare our results with those of other authors that also studied the relationship between T2D and RDW.^12^ In our study, we included just diabetics that already had the disease.

The data that support the findings of this study are openly available in Synapse at https://www.synapse.org/#!Synapse:syn23567519.

### 2.2 Collection of blood samples

Blood samples (20 mL) were collected by intravenous puncture after a night fasting of 8‐12 hours, directly in evacuated tubes (Vacutainer; BD), two containing K3EDTA, for determination of blood count and erythrocytes osmotic stability, and two containing separating gel for the biochemical assays.

### 2.3 Equipment

Mass measurements were made on a precision digital scale (AND, model 870), and volume measurements were made using automatic pipettes (Labsystems; Model Finnpipette Digital). Incubations at 37°C were performed in a thermostated water bath (Marconi; Model MA 184). Centrifugations were performed in a temperature‐controlled centrifuge (Hitachi Koki; model CF15RXII) and absorbance readings on UVU‐VIS spectrophotometer (Hach; Model DR 5000).

### 2.4 Determination of erythrocyte osmotic stability

Osmotic stability of the erythrocyte membrane was determined according to a previously established protocol,^32^ using blood samples as fast as possible, preferably on the same day, taken no more than 12 hours before assay. Blood aliquots of 10 μL were added to a duplicate set of polyethylene tubes (Eppendorf) containing 1.0 mL of 0.0‐1.0 g.dL^−1^ NaCl solution, previously incubated for 10 minutes in the thermostated water bath (Marconi, Model MA‐184) at 37°C. After being hermetically sealed and gently agitated, the tubes were incubated under the same conditions for 30 minutes and then centrifuged at 1600 *g* (Hitachi Koki; Model CFR15XRII) for 10 minutes for supernatant separation and absorbance reading at 540 nm (A540) against water (control) on a UV‐VIS spectrophotometer (Model DR 5000; Hach).

The haemoglobin absorbance at 540 nm (A) was plotted as a function of NaCl (X) concentration, using a sigmoidal regression routine based on the Boltzmann equation,^32^

(1) $$A=\frac{A_{\max}‐A_{\min}}{1+e^{(X‐H_{50})/dX}}+A_{\min}$$

in order to determine the parameter values: Amax and Amin, which represent the mean absorbance values at 540 nm in the maximum and minimum sigmoid plateaus; H_50_, which is the concentration of NaCl that causes 50% of haemolysis; and dX, which represents ¼ of the variation in NaCl concentration responsible for the transition between intact (A_min_) and lysate (A_max_) erythrocytes. The values determined by the programme were accepted only when the statistical adjustment of the curve data was statistically significant (*P* ≤ .05).

The near NaCl concentrations where lysis begins (H0) and ends (H100) were then determined according to the following equations:

(2) $$H_{0}=H_{50}+2dX$$

and

(3) $$H_{100}=H_{50}‐2dX$$

as previously described.^28^

Amin, H_0_, H_50_ and H_100_ are osmotic fragility variables and therefore have inverse relationships with the erythrocyte osmotic stability, but dX is effectively a stability variable; that is, dX increases with increasing osmotic stability of these cells.

### 2.5 Determination of blood count and blood biochemical variables

Routine blood tests were performed at the Clinical Analysis Laboratory of the Clinical Hospital of the Federal University of Uberlandia.

Blood counts were determined using an automated system (Sysmex America Inc., model XN 3.000). Reticulocyte counts were performed visually. The erythrocyte sedimentation rate (ESR) was determined in an automated analysis system (Alifax SPA).

Dosages of total cholesterol (t‐C), HDL cholesterol (HDL‐C), LDL cholesterol (LDL‐C), VLDL cholesterol (VLDL‐C), triglycerides (TGC), glucose (Glu) and C‐reactive protein. (CRP) were made on a Roche automated analyser (Cobas 6000). Glycated haemoglobin (HbA1c) was measured by high‐performance liquid chromatography (BIORAD D‐10).

### 2.6 Statistical analysis

The nature of data distribution in each group was made using the Shapiro‐Wilk test. Data from most of the analysed variables were not normally distributed, and therefore, they were expressed as median (Q1‐Q3), where Q1 and Q3 correspond to the value found in 25% and 75% of the data, organized in an ascending way. The median, or Q2, is the value of half of the data. In a normal distribution, the median is equivalent to the mean, whereas Q1‐Q3 has a similar meaning to standard deviation. The comparison between groups was made using the Mann‐Whitney.

All correlation analyses were performed using the Spearman method as data distribution was not normal for most of the variables studied.

The existence of statistical significance in each analysis was admitted when the *p‐*value was less than .05.

All comparative statistical analyses were performed using SPSS 21 (SPSS Inc, IBM, Chicago, IL, USA). OriginPro 2016 (Microcal) was used to obtain osmotic stability parameters from Boltzmann sigmoid, and R (version 3.6.1 com R studio 1.2.1335 and package corrplot) was employed to make a coloured correlation matrix shown in Figure 3.

## 3 RESULTS

**Figure 2:**
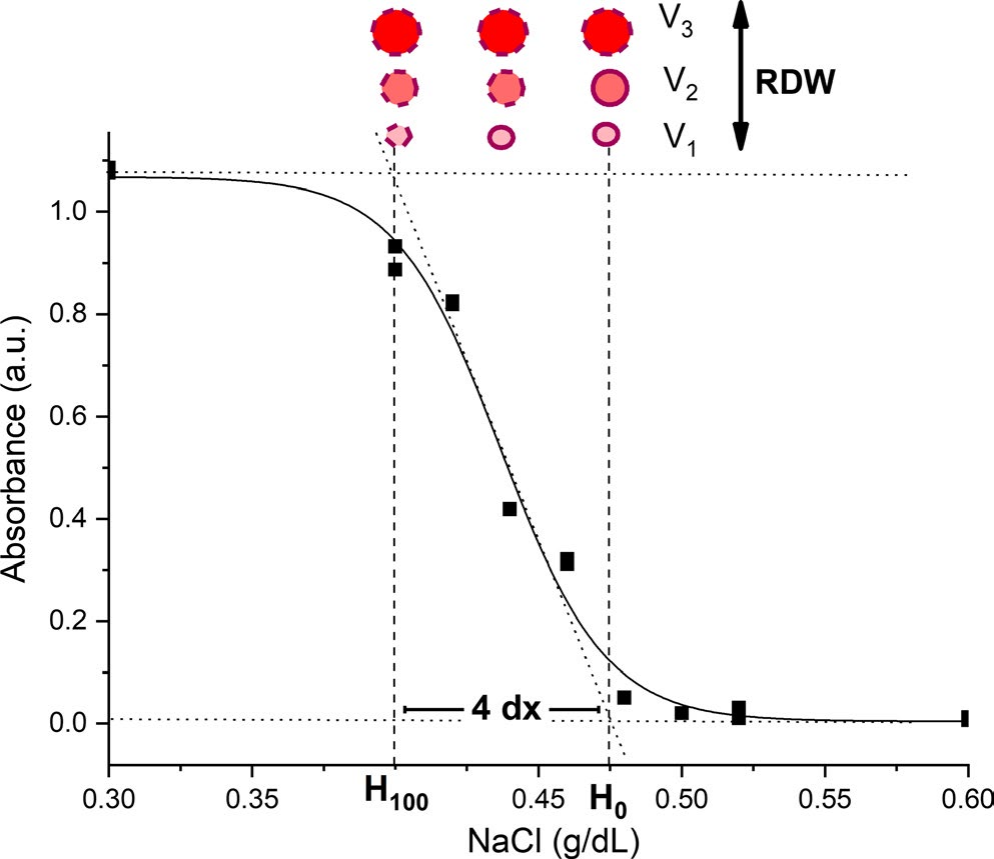
Osmotic fragility curve excerpt of one of the study participants. The interval 4 dX is delimited by H_0_ and H_100_. Reticulocytes are supposed to increase H_0_, whereas microcytic erythrocytes with low MCHC are presumed to decrease H_100_. Thus, both micro‐ and macrocytic erythrocytes will increase both RDW and dX. In the upper side of the figure, the circle represents erythrocytes. Each row of circles represents erythrocytes of the same size in different saline concentrations. The number of rows is correspondent to an increase in RDW. The intensity colour of the circle indicates the haemoglobin concentration, which is expected to lessen insofar as erythrocytes become smaller. Circles with a continuous line around them indicate integer erythrocytes, whereas circles with a discontinuous line represent lysed erythrocytes

Figure 2 shows an interval of a typical erythrocyte osmotic fragility curve used to determine the osmotic fragility and osmotic stability parameters considered in this study. This figure also illustrates the proposed mechanism by the increase of dX from an extension of H_100_ towards lower salt concentrations. According to this idea, the bigger erythrocytes are negatively selected insofar as the size and MCHC decreases.

Table 1 presents the basic characteristics of the groups of volunteers without diabetes and with type 2 diabetes mellitus (T2D), as well as the results of the comparison between the groups of each of the studied variables. Among the differences observed in the diabetic group concerning the non‐diabetic group in both sexes, there were higher RDW values and lower serum iron, transferrin saturation index (TSI), MCHC, MCH and total cholesterol. Men did not show exclusive differences regarding baseline; however, women alone presented higher levels of CRP, leucocyte count and lower MCV.

**Table 1:** Baseline characteristics of non‐diabetic (ND) and diabetic (T2D) female and male^a^

Parameters	Women			Men		
	ND	T2D	*p*	ND	T2D	*p*
	(n = 47)	(n = 32)		(n = 22)	(n = 21)	
Age (years)	58.5 (50.8‐66.3)	62.5 (58‐69.8)	.10	60.5 (44‐67.5)	60.5 (56‐71)	.43
Body mass index (kg/m^2^)	26.3 (24‐28.1)	29.6 (25.7‐32.4)	<.01	27.8 (24.4‐29.5)	28.6 (26.3‐31.8)	.13
Ethnicity^b^						
White	46.8% (22)	53.1% (17)		59.1% (13)	28.6% (6)	
Mixed	31.9% (15)	34.4% (11)		36.4% (8)	42.9% (9)	
Black	21.3% (10)	12.5% (4)		4.5% (1)	28.6% (6)	
Glucose (mmol/L)	5.2 (5‐5.4)	8.2 (6.5‐9.6)	<.01	5.2 (5‐5.6)	7.5 (6.1‐9.9)	<.01
HbA1c (%)	5.4 (5.3‐5.6)	7.9 (6.9‐9.6)	<.01	5.4 (5.4‐5.6)	7.5 (6.6‐8.6)	<.01
Total cholesterol (mmol/L)	4.9 (4.4‐5.6)	4.3 (3.6‐4.9)	<.01	4.9 (4.1‐5.8)	4.3 (3.5‐4.7)	.02
LDL cholesterol (mmol/L)	2.8 (2.3‐3.4)	2.1 (1.6‐2.7)	<.01	2.8 (2.1‐3.7)	2.3 (1.8‐2.9)	.04
HDL cholesterol (mmol/L)	1.4 (1.1‐1.6)	1.2 (1‐1.5)	.08	1.1 (1‐1.4)	1.1 (1‐1.3)	.56
Creatinine (mmol/L)	0.1 (0.1‐0.1)	0.1 (0.1‐0.1)	.77	0.1 (0.1‐0.1)	0.1 (0.1‐0.1)	.76
C‐reactive protein (mg/L)	2.1 (1.1‐4.2)	4.7 (2.1‐9.5)	.02	2.3 (0.9‐5)	3.7 (1.3‐5.8)	.51
Ferritin (nmol/L)	0.4 (0.2‐0.6)	0.2 (0.1‐0.6)	.18	0.5 (0.3‐0.9)	0.5 (0.2‐0.8)	.98
Iron (µmol/L)	16.7 (14.8‐20.8)	12.5 (10.2‐19)	<.01	21.4 (16.2‐24.9)	15.1 (12.4‐20.6)	<.01
Transferrin saturation index (%)	32.8 (25.2‐41.6)	22.5 (18‐27.7)	<.01	38.6 (29.2‐50.2)	29 (22.8‐37)	.04
TIBC (mmol/L)	0.5 (0.5‐0.6)	0.5 (0.5‐0.6)	.11	0.5 (0.5‐0.6)	0.5 (0.5‐0.6)	.50
Haemoglobin (g/dL)	135 (130‐140.5)	132 (126.3‐137.5)	.06	147.5 (142.5‐159)	148 (136‐157.5)	.56
Haematocrit (%)	39.7 (37.4‐41.2)	39.1 (37.6‐40.8)	.42	42.8 (41.5‐45)	42.9 (39.9‐45.4)	.95
RBC (million/mm^3^)	4.6 (4.3‐4.8)	4.6 (4.3‐4.8)	.75	4.9 (4.6‐5.2)	4.9 (4.6‐5.6)	.32
MCV (fL)	86.8 (83‐88.8)	84.5 (80.5‐87.2)	.03	88.5 (84.1‐91.3)	85.6 (81.4‐89.8)	.13
MCH (pg)	29.9 (28.9‐30.8)	28.9 (26.8‐29.7)	<.01	30.8 (29.8‐31.3)	29.3 (27.6‐30.7)	.02
MCHC (mmol/L)	21.4 (20.9‐21.8)	20.9 (20.4‐21.3)	<.01	21.6 (21‐22.2)	21.3 (20.8‐21.8)	.09
RDW (%)	12.8 (12‐13.2)	13.2 (12.6‐14.6)	<.01	12.7 (12.1‐13.2)	13.2 (12.8‐14)	.02
Reticulocyte index (%)	1.1 (0.7‐1.6)	1.2 (0.8‐1.8)	.40	1.1 (0.4‐2.3)	1.3 (0.6‐1.9)	.54
Leucocyte (10^9^ cells/mm^3^)	5.7 (4.7‐6.3)	7 (6‐8)	<.01	6.2 (5.3‐7.3)	6.4 (5.4‐7.3)	.89
Vitamin B9 (nmol/L)	26.2 (20.6‐32.6)	25.5 (20‐31.6)	.93	23.9 (15.8‐31.4)	24.8 (19.8‐30.9)	.40
Vitamin B12 (pmol/L)	279.5 (230.2‐409.8)	275.5 (236.4‐367.6)	.71	307.9 (227.5‐392.6)	296.7 (225.6‐334.6)	.58
Medication^b^						
Statin	12.8% (6)	21.9% (7)		9.1% (2)	14.3% (3)	
Oral hypoglycaemic drugs	19.1% (9)	31.3% (10)		13.6% (3)	19% (4)	
Insulin	2.1% (1)	12.5% (4)		0% (0)	19% (4)	
Anti hypertensives	25.5% (12)	25% (8)		22.7% (5)	66.7% (14)	
Diuretics	8.5% (4)	15.6% (5)		13.6% (3)	4.8% (1)	
Anxiolytics	2.1% (1)	15.6% (5)		9.1% (2)	4.8% (1)	
Antidepressants	4.3% (2)	9.4% (3)		9.1% (2)	9.5% (2)	
Levothyroxine	2.1% (1)	9.4% (3)		9.1% (2)	4.8% (1)	
Others	21.3% (10)	34% (11)		13.6% (3)	14.3% (3)	

Note

As the amount of data in each category was too low, statistical tests between diabetics and non‐diabetics were not performed.

^a^ The comparison between groups was made using the Mann‐Whitney test, with *p‐*values <.05 indicating statistically significant difference.

^b^ Categorical data: percentual (number).

In Table 2, the parameters studied here were compared with individuals grouped into better (HbA1c < 7%) and worse (HbA1c ≥ 7%) glycaemic control for males and females. In the case of women, we found that the volunteers with worse glycaemic control curiously presented lower levels of total cholesterol, although they had higher BMI. Still, in women with worse glycaemic control, it was observed higher values of RDW, dX, reticulocytes, CRP and lymphocytes. However, in this same group, it was also noted lower levels of plasma iron, TSI, haemoglobin, MCH and vitamin D. In both groups male and female, there were higher levels of both glucose and glycated haemoglobin, which was already expected. In men with worse glycaemic control, there were few differences compared to the ones with better glycaemic control. Among these differences, the higher level of ferritin draws the attention; however, there was no difference in CRP between men with worse and good glycaemic control.

**Table 2:** Comparison of studied variables between groups of volunteers with better (HbA1c < 7%) and worse glycaemic control (HbA1c ≥ 7%)

Parameters	Women HbA1c		*p*	Men HbA1c		*p*
	<7% (n = 43)	≥7% (n = 36)		<7% (n = 23)	≥7% (n = 20)	
Age (years)	58.5 (51‐69.5)	61 (58‐68)	.23	63 (54‐68)	58 (45‐72)	.71
Body mass index (kg/m^2^)	26.1 (24.3‐28.7)	27.9 (25.8‐31.5)	.03	27.7 (24.9‐31.1)	28.7 (26.3‐30.6)	.41
Glucose (mmol/L)	5.3 (5‐5.5)	7.6 (5.4‐9.5)	<.01	5.3 (5.1‐5.9)	8.2 (5.3‐11.2)	<.01
HbA1c (%)	5.5 (5.3‐5.9)	8.8 (7.6‐10.4)	<.01	5.5 (5.4‐6.5)	8.5 (7.5‐10.2)	<.01
Total cholesterol (mmol/L)	4.6 (4.2‐5.6)	4.5 (3.9‐5)	.23	4.6 (3.9‐5.7)	4.5 (3.8‐5.1)	.83
LDL cholesterol (mmol/L)	2.7 (1.9‐3.5)	2.4 (1.8‐3)	.19	2.6 (2.1‐3.6)	2.3 (1.9‐2.9)	.22
VLDL cholesterol (mmol/L)	0.6 (0.5‐0.9)	0.5 (0.5‐1)	.35	0.6 (0.4‐0.9)	0.7 (0.5‐0.8)	.65
HDL cholesterol (mmol/L)	1.3 (1.1‐1.6)	1.3 (1‐1.5)	.33	1 (0.9‐1.4)	1.1 (1‐1.4)	.47
Creatinine (mmol/L)	0.1 (0.1‐0.1)	0.1 (0.1‐0.1)	.84	0.1 (0.1‐0.1)	0.1 (0.1‐0.1)	.70
C‐reactive protein (mg/L)	2.3 (0.9‐6.6)	4.2 (1.8‐5.7)	.15	2.6 (1.2‐9.2)	3.2 (1.1‐4.7)	.70
Ferritin (nmol/L)	0.4 (0.2‐0.7)	0.2 (0.1‐0.5)	.17	0.4 (0.2‐0.7)	0.6 (0.5‐1)	.02
Iron (µmol/L)	16.4 (13.7‐21)	13.7 (10.9‐19.3)	.03	19.7 (14.9‐23)	15.3 (14.3‐22.3)	.53
Transferrin saturation index (%)	32.5 (23.5‐42)	24.9 (18.3‐35.7)	<.01	35.5 (25‐48)	30.2 (24.4‐41.7)	.39
Total iron binding capacity (mmol/L)	0.5 (0.5‐0.6)	0.5 (0.5‐0.6)	.15	0.5 (0.5‐0.6)	0.6 (0.5‐0.6)	.16
Haemoglobin (g/dL)	135.5 (130‐140.3)	132 (124‐140)	.05	148 (141‐161)	146.5 (137.8‐157.8)	.50
Haematocrit (%)	39.6 (37.7‐41.2)	39 (35.9‐40.7)	.28	43.8 (41.8‐45.1)	42.2 (39.9‐44.7)	.26
RBC (million/mm^3^)	4.6 (4.3‐4.8)	4.7 (4.2‐4.8)	1.00	4.9 (4.6‐5.2)	4.9 (4.6‐5.5)	.82
MCV (fL)	86.8 (83‐88.7)	84.6 (82.3‐88.1)	.11	87.9 (83.8‐91)	86.3 (82.2‐90.4)	.43
MCH (pg)	29.9 (28.6‐30.8)	29 (27.3‐29.7)	<.01	30.7 (29.2‐31.2)	29.9 (27.9‐31.2)	.36
MCHC (mmol/L)	21.3 (20.9‐21.8)	20.9 (20.6‐21.5)	.03	21.2 (20.8‐22)	21.4 (21.1‐21.9)	.51
Red cell distribution width (%)	12.8 (12.1‐13.3)	13.2 (12.6‐14.2)	<.01	13.2 (12.7‐13.9)	13 (12.3‐13.3)	.19
Reticulocyte index (%)	1 (0.6‐1.5)	1.3 (0.9‐1.9)	.03	1.1 (0.4‐1.9)	1.2 (0.5‐2.2)	.82
Leucocyte count (10^9^ cells/mm^3^)	5.8 (5‐6.7)	6.2 (5.4‐7.4)	.15	5.6 (5.2‐7.3)	6.6 (5.5‐7.5)	.15
Vitamin B9 (nmol/L)	26.3 (21.1‐32)	25.9 (19.4‐35.6)	.92	24.8 (15.8‐31.3)	24.6 (20.9‐26.7)	.85
Vitamin B12 (pmol/L)	262 (228.5‐401)	291.2 (237.2‐448.6)	.32	307.4 (217.4‐375.2)	304.3 (246.1‐339.3)	.80

**Figure 3:**
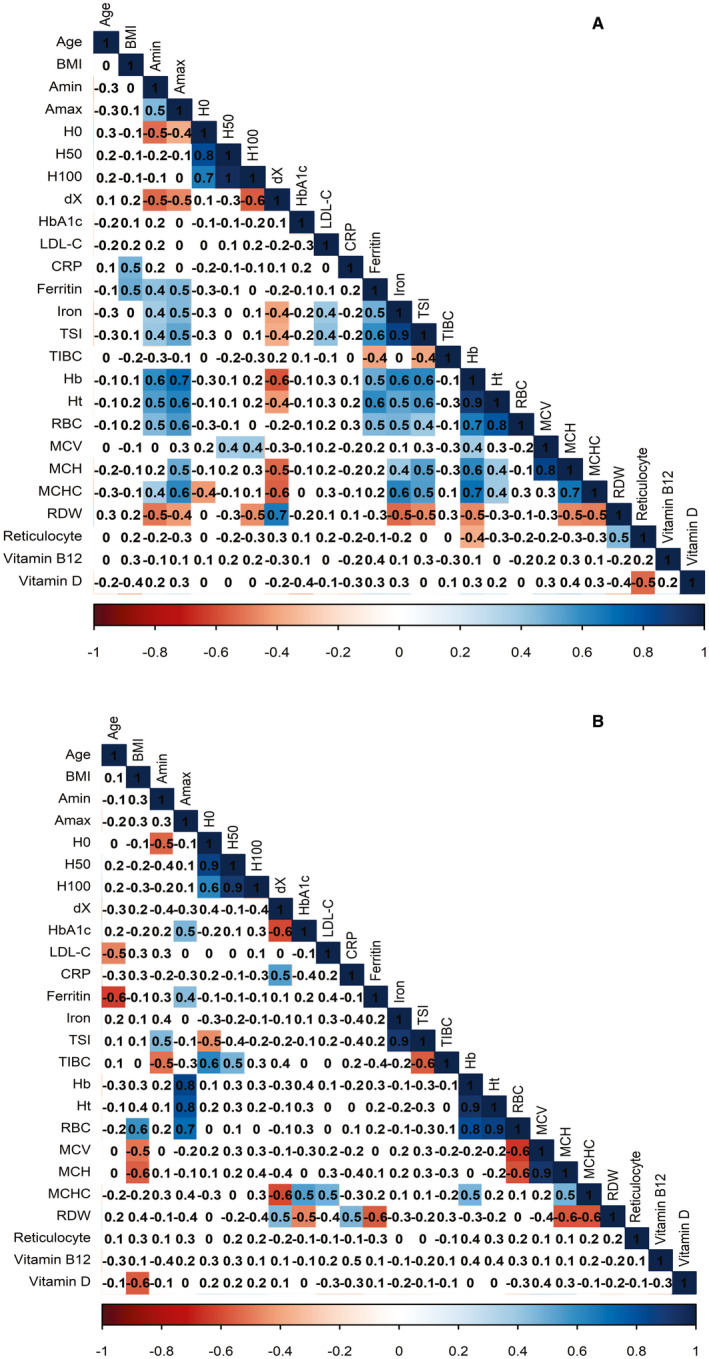
Spearman correlations between pairs of variables studied in the group of diabetic women (A) and men (B). The number within each box represents the Spearman's coefficient (rho) for a specific correlation under consideration. Significant correlations were highlighted by coloured shading, and the colour density is proportional to the correlation strength (rho)

Figure 3 presents the results of the RDW correlation analysis with the parameters studied in the ND and T2D groups. Some of the statistically significant correlations deserve attention. In both sexes and in all or at least most groups, there was positive correlation of RDW with dX and negative correlation of RDW with the following variables: H_100_, MCHC, MCH and ferritin. In the correlations with all or almost all groups, the non‐significant correlations presented p‐values near borderline values, that is *p*  = .1. However, there were many important correlations found just in one of the sexes. For example, RDW presented negative correlation with both Amin and Amax just in women. On the other hand, only in men RDW presented positive correlation with CRP and negative correlation with both glucose and HbA1c.

The main correlations found in both sexes or just in men or women persisted even when adjusted for age and BMI; however, in some cases this procedure resulted in decrease of *p*‐value and correspondent beta value, even though the p‐value was still significant or borderline significant.

## 4 DISCUSSION

### 4.1 RDW in T2D

The occurrence of higher RDW in T2D in relation to ND in both sexes (Table 1) was already expected as high RDW values have been found in the most diverse diseases,^33^, ^34^ especially chronic diseases,^35^ among which stand out type 2 diabetes mellitus.^12^ However, our work is the first one to investigate the possible causes of high RDW in type 2 diabetes. It is possible that our findings also apply to other chronic diseases.

Most studies that have analysed the relationship between RDW and disease are retrospective studies that use test results routinely requested to follow‐up patients with a specific disease. In general, such studies do not have a great control group. Furthermore, the control group in these publications do not usually undergo the same examinations that are performed on the test group. This was the case of the paper done by Nada and colleagues, which found that RDW is higher in diabetics than in non‐diabetics.^12^ Our study was cross‐sectional, and thus, we could include more specific examinations that are not routinely requested in diabetics and even less in non‐diabetics. Such examinations include iron, reticulocytes, vitamins and many others.

It seems that one of the main factors linked to RDW elevation is linked to iron homoeostasis. Indeed, higher RDW values and lower serum iron concentrations were found in diabetic than in non‐diabetic volunteers of both sexes (Table 1). Although iron levels are normal, increased transferrin saturation index (TSI) in both sexes indicates a tendency towards iron deficiency anaemia by inflammation.^36^ Apparently diabetic women are suffering more from lower iron levels, as women with poor glycaemic control, but not men, presented lower values of iron, TSI, haemoglobin, MCH, MCHC and higher RDW, dX, reticulocyte (Table 2).

Other strong evidence of the relationship between RDW increase and iron homeostasis damage comes from the analysis of the correlations in Figure 3. Significant negative correlations were found of RDW with iron, TSI and haemoglobin, but just in females. In practically all groups, however, RDW correlates both with MCH and MCHC.

### 4.2 RDW, iron and CRP

Although type 2 diabetes could be associated with elevated serum iron levels related to undiagnosed hemochromatosis,^37^ in the present study, both the male diabetic group and female diabetic group had lower iron levels compared to the non‐diabetic counterpart (Table 1). Furthermore, women with worse glycaemic control, that is HbA1c higher than 7, also presented lower levels of iron (Table 2). However, it should be noted that the observed values do not characterize anaemia as they are within the serum iron reference range, which is 33‐193 µg/dL. Despite that, the lower iron concentrations found in diabetic women indicate a tendency towards iron deficiency anaemia, even because transferrin saturation is lower in the diabetic women group than in the other groups (Table 1).

One possible explanation for the lower levels of iron in our diabetic patients could be an exacerbation of inflammation, as elevated values of C‐reactive protein (CRP) were found and leucocytes in women with T2D (Table 1). There is also indication that diabetic men had considerable inflammation, as the ferritin was increased in males with poor glycaemic control (Table 2). In inflammation, the serum iron is diminished because of both reduced absorption and increased storage.^38^ It is improbable that the lower level of iron was because of malnourishment, as levels of vitamins were normal and without differences among the groups (Table 1).

C‐reactive protein had a curious behaviour in relation to sex. It was increased in diabetic women in relation to non‐diabetic women but presented positive correlation with RDW just in men. However, there is indication of positive association of CRP and RDW in both sexes. It is in concordance with authors that showed involvement of inflammation in higher RDW values.^39^

### 4.3 RDW, iron, MCV and reticulocytes

The mean corpuscular volume (MCV) value of diabetic women was smaller than in non‐diabetic women (Table 1), possibly due to the tendency to microcytosis driven by lower iron levels in the female diabetic group (Table 1) and in women with poor glycaemic control (Table 2). It is an interesting result, as other authors have reported that erythrocytes from diabetics are bigger than those from non‐diabetics.^40^, ^41^, ^42^, ^43^ These findings were obtained either by Atomic Force Microscopy^40^, ^41^ or automated haematological analyser by the Coulter method,^42^, ^43^ like our case.

The discrepancy between the MCV value we found in diabetics in relation to scientific literature probably occurred because our patients in both groups were treated for vitamin B12 deficiency, especially those treated with metformin, whose risk of presenting a vitamin B12 deficiency is higher.^44^, ^45^ The treatment with vitamin B12 probably masked signs of a tendency towards iron deficiency. If the patients were not treated, possibly, the RDW in diabetics would be even higher. One could ask: What if the diabetic patients receive iron supplementation? We think it would not have an effect over RDW, as the increased levels of both ferritin and CRP in diabetics suggest that the lower iron in diabetics were due to an inflammatory state.^36^, ^46^ Furthermore, the positive correlation observed between RDW and reticulocyte index in diabetic women (Figure 3) supports the contribution of reticulocyte increase in RDW elevation.

The increase of reticulocytes probably resulted from a higher glucose media, which decrease the lifespan of erythrocyte with a resultant increase of reticulocytes.^5^, ^47^, ^48^ In our study, it could be inferred by higher value of reticulocytes in females with poor glycaemic control (Table 2).

Men probably did not present lower levels of MCV because diabetic males had significantly higher levels of iron than diabetic females (*P* = .042). The same occurred for non‐diabetic males in relation to non‐diabetic women (*P* = .038). The increased levels of iron in both diabetic and non‐diabetic men compared to the women counterpart do not seem to be a consequence of diabetes. Naturally women require more iron than men because of menstruation and pregnancy.^49^ However, most women in the present study were in post‐menopause.

Another possible tendency to macrocytosis is metformin usage, which was used by 23% of diabetic females and by 14.3% of diabetic males. It is known that metformin is associated with vitamin B12 deficiency.^44^, ^45^ However, there was no difference in the levels of this vitamin between the groups in the present study (Table 1), at least in part due to prophylactic supplementation in vegan patients and those with blood levels of the vitamin in the lower limit of the reference range. Other medications also commonly used by diabetics, such as statins, hypotensors, aspirin and even insulin, does not seem to have an effect on MCV as we have not found papers reporting it in vivo in people with T2D. However, more studies should be done to clarify this question.

In the population studied here, the RDW increases under the simultaneous influence of a factor associated with volume increase (reticulocyte) and other factors associated with volume decrease (lower iron levels).^50^ As RDW represents heterogeneity in the volume distribution of erythrocytes, this explains why, then, RDW was not correlated with MCV (Figure 3), although a positive correlation between those variables was reported in patients who underwent bariatric surgery^29^ and a negative correlation has been found in pregnant women with pre‐eclampsia.^28^

### 4.4 RDW, MCHC, cholesterol and osmotic stability

Lower MCH and MCHC values were found in diabetics compared to non‐diabetics (Table 1), certainly due to the lower iron levels found in that group. Lower MCHC values would result in greater erythrocyte osmotic stability, because of the lowering of internal osmotic pressure.^27^ However, the parameters of osmotic stability were not significantly different between the studied groups (Table 1). This lack of difference in osmotic stability between the groups could, at least in part, be justified by the antagonistic influence of factors affecting this erythrocyte property. On the one hand, lower MCHC values would tend to promote increased osmotic stability.^27^ On the other hand, lower levels of total cholesterol and LDL cholesterol (LDL‐C) in the diabetic group (Table 1) would contribute to decreased osmotic stability.^51^ However, it is noteworthy that there is an increased dX in women with poor glycaemic control (Table 2), indicating that, in fact, erythrocyte osmotic stability really tends to be increased in diabetes, at least in women.

The lower LDL‐C levels in both diabetic women and men do not seem to have a desirable meaning, as one might infer as lower LDL‐cholesterol levels were associated with poor glycaemic control (Table 2). These lower total cholesterol and LDL‐cholesterol levels observed in the diabetic group usually are not a primary characteristic of the disease,^52^ in part by an increased synthesis of cholesterol in T2D.^53^ Apparently, low level of cholesterol in our diabetic patients could be a consequence of cholesterol‐lowering drugs, such as statins.^54^ However, performed statistical analysis without statin‐using participants still indicated lower levels of cholesterol in the diabetic group of both sexes. Due to its clinical relevance, it is a question that deserves further investigation.

In the bloodstream, erythrocytes interact with lipoproteins, so they can receive or deliver cholesterol to these structures.^55^ Thus, much lower levels of LDL‐C in diabetic women would lead to lower cholesterol levels in the erythrocyte membrane, which would lead to lower osmotic resistance, as cholesterol makes the erythrocyte less rigid membrane^56^, ^57^, ^58^ and thus more resistant to swelling due to water entry when in hypotonic medium.^56^

Some authors found positive relation between high cholesterol levels in erythrocyte membranes associated with higher RDW values in coronary artery disease patients.^59^ Unfortunately, the membrane cholesterol content was not evaluated in this study, but was presumably lower in diabetic volunteers, as they presented lower levels of LDL cholesterol. It indicates that cholesterol is not a good predictor for RDW values, as cholesterol can be increased or decreased according to the disease, whereas cholesterol tends to be increased.

### 4.5 RDW, dX and H_100_

Although the osmotic stability parameters were not different among the groups, there was important correlation of RDW with both dX (positive) and H_100_ (negative) in practically all groups. The dX by itself is not enough to indicate whether osmotic stability has increased or decreased. According to our hypothesis shown in Figure 2, reticulocytes would increase dX by increasing values of H_0_, whereas small erythrocytes with low MCHC would increase dX by decreasing values of H_100_. Thus, the negative correlation between dX and H_100_ (Figure 3) is not surprising. Correlations involving osmotic stability parameters like dX and its boundaries (H_0_ and H_100_) are very scarce in the literature. Some correlations containing these parameters are just present in more recent publications of our research group, specifically in a study with pregnant women.^27^, ^28^, ^60^ However, this is the first time that a solid hypothesis is made to clarify the positive correlation between RDW and dX (Figure 2), although this correlation has also been found in a study comprehending healthy subjects.^60^ Essentially, with few exceptions indicated along the text, all correlations which have stability parameters appear here for the first time.

The association of RDW with dX, not only in the diabetic group but also in the other groups (Figure 3) shows that dX has an equivalent meaning to that of the RDW in expressing heterogeneity in the distribution of erythrocyte population volumes (Figure 2). This similarity certainly comes from the fact that an increase in the amount of smaller erythrocyte, is due to the tendency of an iron deficiency anaemia,^38^ which is responsible for both the elevation of RDW and osmotic resistance. The RDW would rise because these smaller erythrocytes enhance the volume difference among erythrocytes. On the other hand, these smaller erythrocytes are also hypochromic; that is, they have lesser haemoglobin content, resulting in an increase of osmotic resistance.^27^

Interestingly, dX showed significant negative correlations with MCHC in both sexes (Figure 3). This means that the increase in erythrocyte stability is being driven by the decrease in haemoglobin concentration in erythrocytes, certainly due to decreased osmotic pressure.^28^, ^61^ Indeed, this corroborates the negative correlation observed between RDW and H_100_ in the diabetic women group (Figure 3), as H_100_ represents the lowest saline concentration in which the most osmotically resistant erythrocytes will suffer lysis. The negative correlation of dX with MCHC shows that these osmotically more resistant erythrocytes are probably those with lower haemoglobin content.

The negative correlation of RDW with both HbA1c and glucose in diabetic men is very intriguing, and there is not a simple answer for that. For example, Engstrom and colleagues found positive correlation between RDW and HbA1c in both male and female diabetics, but negative correlation between RDW and glucose.^62^ Other authors also found positive correlation between RDW and HbA1c, but in non‐diabetics.^63^, ^64^ However, concerning diabetes, some authors, like us, found negative correlation between RDW and HbA1c,^65^ whereas other authors also studying T2D did not find any correlation between these two variables neither in male nor in female.^66^ Thus, there is no consensus about the true direction of the correlation between RDW and HbA1c in diabetes yet.

Engstrom et al^62^ explained their results based on the short duration of erythrocytes in glucose rich media, as characteristic of T2D. However, this hypothesis better explains the negative correlation of RDW and glucose, in accordance with our results. In our study, contrary to that of Engstrom and colleagues, the correlations of RDW with both HbA1c and glucose had the same direction, as both were negative. However, such correlations were restricted to the diabetic men group. We hypothesized that our results regarding these two correlations could be explained by increase of reticulocytes as a result of reduction of erythrocyte lifespan.^67^

### 4.6 Limitations and perspectives

Unfortunately, this study did not evaluate membrane cholesterol content, which certainly represents an important limitation. But surely this is a question that deserves further exploration as the evident equivalence of meaning between RDW and dX suggests that this osmotic stability variable may also have the same predictive power that has been attributed to RDW. Another important limitation is the lack of a test for erythropoietin.

Our study would be even better if we had measured the erythrocyte lifespan. Some studies have shown that older erythrocytes present decreased osmotic resistance.^68^, ^69^ It is in accordance with our study, as we have signs that the erythrocyte lifespan was decreased, with a lower number of older erythrocytes, as indicated by the increase in reticulocytes. Thus, whereas old erythrocytes decrease osmotic resistance, the young ones, especially reticulocytes, would increase the osmotic resistance. The measure of erythrocyte ageing was not performed because of technical limitations.

The small number of men in the study can also be considered a limitation, although, for some authors, an amount of about 10 individuals would be enough to perform a correlation.^70^ Others propose a sample size much higher, especially in the case of the Spearman correlation. In general, the higher the correlation coefficient, the lower the sample necessary.^71^ However, a correlation coefficient above 0.7 can be a sign of collinearity and deserve attention.^72^ Thus, even a very high correlation coefficient can be useless, even with a sufficient sample size. Otherwise, many statistical values are based on conventions, as the own value of significance.

## 5 CONCLUSION

In the present study, the increase in red cell distribution width (RDW) observed in women and men with type 2 diabetes mellitus occurred mainly by decreased iron levels in both groups. The iron lowering presumably resulted in lower MCHC and, consequently, an increase in osmotic stability. Decreasing of MCV should be expected, but that certainly did not happen because of increased reticulocyte index as a result of reduction of erythrocyte lifespan due to high glucose media typical of type 2 diabetes.

As RDW was positively correlated with dX and negatively with H100 in virtually all studied groups, it indicates that an increase of RDW is related to an increase of erythrocyte osmotic stability independently of the presence of type 2 diabetes. Thus, both dX and H_100_ could be considered intrinsic properties of erythrocytes.

Our results suggest that RDW has a similar meaning to dX, especially because of small hypochromic erythrocytes, which are due to the tendency towards iron deficiency anaemia. However, the increase of both RDW and osmotic resistance will be driven mainly by small erythrocytes, with lower MCHC.

## CONFLICT OF INTEREST

The authors confirm that there are no conflicts of interest.

## AUTHOR CONTRIBUTIONS

**Maria Aparecida Knychala:** Conceptualization (equal); Data curation (equal); Investigation (lead); Project administration (equal); Resources (lead); Visualization (equal); Writing‐original draft (equal); Writing‐review & editing (equal). **Mario da Silva Garrote‐Filho:** Conceptualization (equal); Data curation (equal); Formal analysis (lead); Investigation (lead); Project administration (equal); Software (lead); Supervision (lead); Validation (equal); Visualization (equal); Writing‐original draft (equal); Writing‐review & editing (lead). **Breno Batista‐da‐Silva:** Data curation (equal); Formal analysis (equal); Investigation (equal); Software (equal); Writing‐original draft (equal). **Samantha Neves‐de‐Oliveira:** Data curation (equal); Investigation (equal); Writing‐original draft (equal). **Sarah Yasminy‐Luz:** Investigation (equal); Writing‐original draft (equal). **Manuela Ortega Marques‐Rodrigues:** Investigation (equal); Writing‐original draft (equal). **Nilson Penha‐Silva:** Conceptualization (lead); Funding acquisition (lead); Investigation (equal); Methodology (lead); Project administration (equal); Supervision (equal); Validation (equal); Visualization (equal); Writing‐original draft (equal).

## Data Availability

The raw data used in this study have been deposited in Synapse (https://www.synapse.org/#!Synapse:syn23567519), and the data are available from everyone registered on Synapse. The register is free.
